# Supplementary material for: MetaRibo-Seq measures translation in microbiomes
Source: Nat Commun. 2020 Jun 29;11:3268. doi: 10.1038/s41467-020-17081-z (PMC7324362; doi:10.1038/s41467-020-17081-z)
Supplement: Supplementary file 10 — Supplementary Data 7 [file 41467_2020_17081_MOESM10_ESM.zip › File2/Confidence_High_Taxonomy/241043_out.krona.html]

Javascript must be enabled to view this page.

members
magnitude
magnitudeUnassigned
count
unassigned
taxon
rank

241043\_out

4

superkingdom
4
2

4
phylum
976

200643
4
class

171549
order
4

family
4
171552

838
4
genus

4
species

SRS011134\_contig\_number\_36071SRS053398\_contig\_number\_23058SRS103987\_contig\_number\_1194SRS144603\_contig\_number\_14499
165179
